# Aging, Emotion, Attention, and Binding in the Taboo Stroop Task: Data and Theories

**DOI:** 10.3390/ijerph121012803

**Published:** 2015-10-14

**Authors:** Donald G. MacKay, Laura W. Johnson, Elizabeth R. Graham, Deborah M. Burke

**Affiliations:** 1 Department of Psychology, University of California, Los Angeles, CA 90095, USA; E-Mail: laurajohnson@ucla.edu; 2 Department of Psychology, Pomona College and Claremont Graduate University, Claremont, CA 91711, USA; E-Mails: elizabeth.graham@pomona.edu (E.R.G.); dburke@pomona.edu (D.M.B.)

**Keywords:** aging, emotion, attention, memory, taboo Stroop task, binding theory, transmission deficit hypothesis, resource capacity theory, socio-emotional selectivity theory, inhibition deficit theory

## Abstract

How does aging impact relations between emotion, memory, and attention? To address this question, young and older adults named the font colors of taboo and neutral words, some of which recurred in the same font color or screen location throughout two color-naming experiments. The results indicated longer color-naming response times (RTs) for taboo than neutral base-words (*taboo Stroop interference*); better incidental recognition of colors and locations consistently associated with taboo *versus* neutral words (*taboo context-memory enhancement*); and greater speed-up in color-naming RTs with repetition of color-consistent than color-inconsistent taboo words, but no analogous speed-up with repetition of location-consistent or location-inconsistent taboo words (*the consistency type by repetition interaction for taboo words*). All three phenomena remained constant with aging, consistent with the transmission deficit hypothesis and binding theory, where familiar emotional words trigger age-invariant reactions for prioritizing the binding of contextual features to the source of emotion. Binding theory also accurately predicted the interaction between consistency type and repetition for taboo words. However, one or more aspects of these phenomena failed to support the inhibition deficit hypothesis, resource capacity theory, or socio-emotional selectivity theory. We conclude that binding theory warrants further test in a range of paradigms, and that relations between aging and emotion, memory, and attention may depend on whether the task and stimuli trigger fast-reaction, involuntary binding processes, as in the taboo Stroop paradigm.

## 1. Introduction

This study addresses a timely and theoretically significant question in psychology and the field of cognitive aging: How does aging impact relations between emotion, memory, and attention? The timeliness reflects two recent developments. One is the emergence of detailed theories of emotion, memory, and attention that are sufficiently general to encompass cognitive aging. The other is development of an experimental paradigm well suited for testing those theories. We begin by describing this “taboo Stroop” paradigm and the phenomena demonstrated to date through its use. We then outline two general theories of emotion, memory, and attention and their predictions for the present experiments. 

### 1.1. The Taboo Stroop Paradigm

On each trial in the taboo Stroop task, participants attend to and name the font color of a word as quickly as possible—with instructions to ignore the meaning of the word, its screen location, and the possibility that it might be taboo. The concept of attention is well-defined using these procedures—a major advance over the intuitive definitions of attention adopted in other paradigms. Because font color is the basis for correct responses in the Stroop task, participants who respond correctly must have attended to the word’s font color. The attended dimension is therefore font color, and variations in color-naming response times (RTs) relative to a neutral or baseline condition can be said to influence attention to the font color as well as to response selection in the taboo Stroop task. One source of influence is emotional arousal because color-naming RTs in this task are reliably longer for taboo than neutral words (controlled for length, familiarity, complexity, and set size)—a phenomenon known as *taboo Stroop interference* (e.g., [1,2]). 

Note that “emotional arousal” and “emotion” are also well-defined in this paradigm because taboo words reliably increase skin conductance, the *sine qua non* signature of emotional arousal (see, e.g., [3]). This contrasts with paradigms that use “emotion words” as stimuli (*i.e.*, words such as *fear* and *sadness* that name emotions). Such words don’t really qualify as “emotional” or “arousing” because they do not consistently trigger galvanic skin responses, and people can discuss *fear* or *sadness* without becoming fearful or sad.

Finally, note that unattended dimensions in the taboo Stroop task are also well-defined. For example, word meaning is unattended in taboo Stroop tasks because participants are instructed to ignore that dimension and when they respond with the word rather than its font color, that trial is discarded in RT analyses. Contextual features such as the screen location of Stroop stimuli likewise count as unattended under this operational definition—a feature that allowed MacKay and Ahmetzanov [4] to demonstrate effects of attention on memory by comparing performance in surprise tests for the font color (attended) *vs.* the screen location (unattended) of words in the color-naming task. 

These incidental memory tests have contributed significant insights into what happens in the taboo Stroop paradigm. For example, when participants free recalled as many words as possible after naming their font colors, MacKay *et al.* [2] and MacKay and Ahmetzanov [4] observed *taboo event-memory enhancement*: better incidental recall of taboo than neutral words. When participants were asked to recall contextual features (font color and screen location) that consistently accompanied some of the words during color naming, MacKay *et al.* also observed *taboo context-memory enhancement:* better incidental recognition of font colors and screen locations consistently associated with taboo than neutral words (for an analogous effect using non-taboo emotional words, see [5]). Both of these effects have real world parallels in the phenomenon known as flashbulb memories (see, e.g., [4,6,7,8,9,10]). 

### 1.2. The Present Study in Overview

The present study consists of two experiments with the same goal: to test general theories of emotion, attention, aging, and memory as applied to taboo Stroop interference (longer color-naming RTs for taboo than neutral words) and taboo context-memory enhancement (better incidental recall of font colors and screen locations consistently associated with taboo than neutral words during color naming). In Experiment 1, young and older adults named font colors with half the words in the standard color-inconsistent condition (where words recur in different font colors throughout the experiment) and the remaining half in the color-consistent condition (where each word recurs in the same font color throughout the color-naming task). Young and older participants in Experiment 2 also named font colors, but half the words were location-inconsistent (or recurred in different screen locations throughout the experiment), and half were location-consistent (or recurred in the same screen location throughout the color-naming task). There were three dependent variables: color-naming RTs (Experiments 1 and 2), recognition memory for the font color of color-consistent words (Experiment 1), and recognition memory for the screen location of location-consistent words (Experiment 2). Prior studies using the taboo Stroop task have examined effects of neither normal cognitive aging nor color- and location-consistency nor word repetition on either color-naming RTs or recognition memory for context. 

However, prior studies have examined how word repetition *per se* influences the color-naming RTs of young adults in the standard color-inconsistent condition of the taboo Stroop task. The well-established finding is habituation: Color-naming RTs remain relatively constant as neutral words repeat but gradually decrease for taboo words due to habituation with repetition of the emotional reactions to taboo words (see, e.g., [2]). This speed-up in color-naming RTs as taboo words repeat reflects two types of habituation: *generalized* habituation, which occurs when *any* taboo word repeatedly elicits an emotional reaction, perhaps because, despite warnings and practice trials involving taboo words, repetition diminishes the initial shock of seeing any taboo-category word whatsoever in a university-sponsored experiment; and *word-specific* habituation, which occurs when a *particular* taboo word repeatedly elicits its unique constellation of emotional reactions (see [2]). 

However, both types of habituation are logically independent of the current focus: the effect of emotion on the ability to link a repeated word to its differing contexts of occurrence in the color-consistent *versus* location-consistent conditions. Regardless of the specific context-of-occurrence being examined, habituation to taboo words can be expected to have constant effects. Thus, if manipulating these contextual variables yields different results, habituation cannot, by itself, explain those results. In short, the current study dissociates general and specific habituation to taboo words from the effect of emotion in linking a recurring stimulus with its context-of-occurrence.

### 1.3. General Theories of Emotion, Memory, and Attention

Comparing effects of repetition on memory and color-naming RTs in the color-consistent condition (Experiment 1) *versus* location-consistent condition (Experiment 2) is important for discriminating between two general theories of emotion, memory, and attention: resource capacity theory (RCT) *versus* binding theory. Before addressing aging, we first review how these theories explain taboo Stroop interference and taboo context-memory enhancement. We then outline their predictions for how repetition will impact color-naming RTs and recognition memory in the contrasting consistency conditions in Experiments 1 *versus* 2. 

#### 1.3.1. Resource Capacity Theory (RCT)

Under RCT, emotional stimuli such as taboo words attract limited capacity attentional resources, thereby reducing available resources for processing concurrent neutral stimuli (see, e.g., [11]). This RCT assumption readily explains taboo Stroop interference: Taboo words attract more attentional resources than neutral words, and, given limited attentional capacity, this means that taboo words will reduce available resources for responding to the font color, thereby lengthening color-naming RTs for taboo relative to neutral words—the taboo Stroop interference effect. 

By adding an additional, widely-held assumption, RCT can also explain taboo context-memory enhancement. Under this assumption, the extra resources devoted to an emotional stimulus enable the formation of an image that includes the emotional stimulus and its physical context-of-occurrence (see, e.g., [6,7,8,9,10]). With images formed for taboo but not neutral stimuli in the taboo Stroop task, memory for font color and screen location will be better for taboo than neutral words.

Turning to the different consistency type conditions in Experiments 1 and 2, RCT predicted the same degree of facilitation for color-naming RTs when taboo word-color combinations repeat and when taboo word-location combinations repeat. On initial exposure to a taboo word-color combination in Experiment 1 or word-location combination in Experiment 2, color-naming RTs will be slow because creating the image of a taboo word in its new font color or screen location consumes attentional resources under RCT, and RTs will remain slow as color- and location-inconsistent taboo words repeat because taboo words in continually changing color- and location-contexts will call for the formation of new images on each trial. 

However, RCT predicted a sharp decrease in RTs when taboo words repeat in the color- and location-*consistent* conditions: The reason is that when the *same* taboo word re-appears in the *same* color- or location-context, this word-in-context image can be quickly retrieved from memory without being formed anew—which will free up attentional capacity for quickly naming the font color of the word. 

Conversely, RCT predicted no sharp drop in RTs as color- or location-consistent neutral words repeat in the two experiments. The reason is that neutral words neither consume attentional capacity nor form enduring images under RCT, so that color-naming RTs will be fast and invariant in the consistent- and inconsistent-context conditions when neutral words repeat in Experiments 1 and 2. 

#### 1.3.2. Binding Theory

Under the binding theory of emotion, memory, and attention, taboo words activate representational units (called content nodes or nodes for short) with direct links to an emotional reaction system (in the amygdala)—which triggers emotional reactions (including increased skin conductance)—the basis for the higher arousal ratings for taboo than neutral words in taboo Stroop tasks. Links from the emotional reaction system to the system of binding nodes (in the hippocampal region) serve to prioritize competing binding processes. Binding processes compete whenever a single node must be bound to more than one node, which occurs in taboo Stroop tasks when the node representing the font color of a taboo word must become bound to the color name response and to the source of emotion (here, the node representing the meaning of the taboo word). 

Competing binding processes are prioritized via the priority binding principle: *A neutral binding process becomes delayed until an emotion-linked binding process involving the same unit has been completed* (see [2,12]; see [13] for explanations of a variety of additional perception and memory findings under a similar theory of emotion-linked priority binding) [14]. 

#### 1.3.3. Priority Binding in Taboo Stroop Tasks: Predictions Illustrated

**Figure 1:**
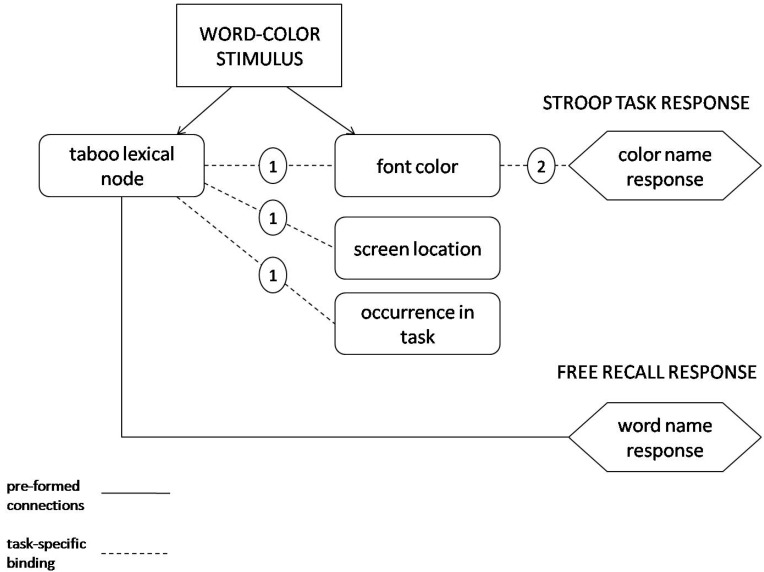
Links involved in naming the font color of taboo words and recalling their occurrence-in-the-task under binding theory. When a taboo word-color stimulus is presented, preformed connections (solid lines) enable activation of two nodes: the lexical node for the taboo word and a node representing its font color. Binding processes then form four task-specific links (broken lines): a link between the font color and the lexical node for the taboo word, a link between the font color and the color-naming response, a link between the lexical node for the taboo word and its screen location, and a link between the lexical node for the taboo word and nodes representing its episodic occurrence in the task. The circled numbers indicate the order of occurrence of these binding processes. Note that under the priority binding principle, font color first becomes bound to the taboo lexical node (1) and then to the color-naming response (2), which is the basis for taboo Stroop interference under binding theory.

Under binding theory, initial presentation of a word-color stimulus automatically activates four types of nodes illustrated in Figure 1: a node representing the font color of the word, a node representing its screen location, a node representing its episodic occurrence in the task, and the lexical node for the word, which is the lowest level representation of word meaning under binding theory. In taboo Stroop tasks, the lexical node for taboo words is also the source of emotion—triggered via a direct link to the emotional reaction system (not shown in Figure 1) [15]. 

Activating a font color node does not, of course, automatically activate a word or color name response: Normally, people neither say aloud printed words that they see nor name the many colors that they routinely encounter in everyday scenes. Note, however, that the font color of taboo words in the taboo Stroop task engages two conflicting binding processes: an emotion-linked one for connecting the lexical node for the taboo word to its font color, and a neutral one for connecting the font color of the taboo word to the appropriate color name response (see Figure 1). Now, the priority binding principle resolves this conflict by assigning priority to the emotion-linked binding process, and delaying binding of the font color to its color name response, which lengthens RTs for taboo words relative to neutral words—the basis for taboo Stroop interference under binding theory (see, e.g., [4]). The numbered labels for the links shown in Figure 1 illustrate this conflict resolution sequence for a taboo lexical node. 

Turning to taboo context-memory enhancement, neutral contextual features such as font color and screen location become bound with priority to the lexical node for taboo but not neutral words in binding theory (see Figure 1). The priority binding principle therefore explains taboo context-memory enhancement as reflecting greater likelihood of encoding font color and screen location for taboo than neutral words. 

Turning to the relation between word repetition and consistency, binding theory predicts a sharp decline in color-naming RTs with repetition of color-consistent but not color-inconsistent taboo words in Experiment 1. The reason is that a taboo lexical node in the color-consistent condition will become bound to its font color on the first few trials, so that new font-color binding becomes unnecessary on subsequent trials as the same word-color combination recurs. With priority binding of font color for taboo but not neutral words as its basis, taboo Stroop interference will therefore be robust on initial trials, but diminish sharply as color-consistent taboo words repeat. However, Stroop interference will be robust on initial *and* subsequent trials for color-inconsistent taboo words, which must become bound to their new font color on each trial. 

In contrast, binding theory (unlike RCT) predicts no difference in color-naming RTs for location-consistent *vs.* location-inconsistent taboo words as a function of repetition. Like color-consistent taboo words, location-consistent taboo words become bound to their location on the first few trials, rendering location binding unnecessary when the same word-location combination recurs on subsequent trials. However, different nodes represent screen location *vs.* font color (see Figure 1), so that location-context binding (unlike color-context binding) will neither compete with nor delay the binding of a taboo word to its font color—the basis for taboo Stroop interference. Taboo Stroop interference will therefore remain robust with repetition of location-consistent and location-inconsistent taboo words under binding theory. 

### 1.4. Aging, the Taboo Stroop Task, and Binding Theory

Prior studies provide mixed evidence that negative emotional stimuli enhance context-memory and/or the processing of attended dimensions differentially for older relative to young adults (see the meta-analysis in [16]). Possible reasons are that most of these studies neither tested for effects of arousal nor reported the arousal levels of their stimuli, which were “emotional” words (e.g., *danger*) that are known to elicit weak emotional reactions at best—an especially serious problem for testing some of the predictions described below. This was a major reason the present studies adopted the taboo Stroop task: Taboo words are associated with very strong emotional reactions (see MacKay *et al.* [2]). 

Binding theory incorporates the Transmission Deficit Hypothesis (TDH) for explaining age-linked effects of emotion, as well as general slowing, new learning deficits, and the relative age-invariance of implicit memory in cognitive aging (see [17]). Under the TDH, connections between frequently used representations remain intact with aging ([18,19]), and the connections underlying comprehension, emotional reactions, and priority binding for taboo words receive frequent use in everyday life. First, comprehension of taboo and neutral words rated as highly familiar by young and older adults engages frequently used connections that will remain invariant with aging under TDH. Second, connections for triggering emotional reactions to familiar taboo words receive equally extensive practice and will likewise remain invariant with aging under TDH. Third, the priority binding principle receives even more extensive use than these word-specific connections in everyday life, and will also remain invariant with aging under TDH. Binding theory therefore predicted age-invariance for taboo Stroop interference and taboo context-memory enhancement. 

Unlike binding theory, other theories (RCT, inhibition deficit theory, and socio-emotional selectivity theory) predict age effects in the taboo Stroop task, but for didactic reasons, we reserve description of these complex predictions for the General Discussion section. 

## 2. Experiment 1: Aging and Effects of Color Consistency

Young and older adults in Experiment 1 named the font color of words in two randomly intermixed conditions: the standard color-inconsistent condition (where words recurred six times in a different font color each time), and the color-consistent condition (where words recurred six times in the same font color throughout the task). They then received a surprise color-recognition test for the color of the color-consistent words shown during color naming. 

RCT and binding theory predicted taboo Stroop interference (*i.e.*, longer color-naming RTs for taboo than neutral words), context-memory enhancement (*i.e.*, superior memory accuracy and confidence in recognizing the font color of taboo than neutral words in the color-consistent condition), a sharp decrease in color-naming RTs as taboo words repeat in the color-consistent but not color-inconsistent condition, and no sharp decline in color-naming RTs with repetition of color-consistent neutral words. Finally, binding theory predicted no age differences in any of these effects. 

### 2.1. Method

Instructions informed participants that they would see words that might be considered offensive or obscene and that they could stop the experiment without penalty for any reason at any time, but none chose that option.

#### 2.1.1. Participants

Participants were 40 young and 40 older adults with background characteristics and statistics shown in Table 1. All were native English speakers, reported good health, lived at home, and passed the Ishihara color blindness test [20]. All participants gave informed consent for inclusion before participating in the study. The study protocol was approved by the Institutional Review Board at Pomona College.

**Table 1:** Background characteristics of participants in Experiments 1 and 2.

Variable	Young	Older
Experiment 1 (N = 40 young, 40 older)		
Age	19.18 (1.13) ^a^	71.95 (5.42) ^b^
Percentage female	70.0% ^a^	82.5% ^a^
Shipley vocabulary score	33.72 (3.48) ^a^	36.15 (3.35) ^b^
Years of education	13.23 (1.27) ^a^	17.10 (2.66) ^b^
Visual acuity (Snellen denominator)	21.50 (2.82) ^a^	34.75 (9.80) ^b^
Minimum Mini-Mental State Test score		27
Experiment 2 (N = 40 young, 40 older)		
Age	20.45 (1.24) ^a^	72.95 (6.05) ^b^
Percentage female	65.0% ^a^	72.5% ^a^
Shipley vocabulary score	32.20 (3.59) ^a^	35.63 (4.01) ^b^
Years of education	14.46 (1.24) ^a^	16.00 (3.76) ^b^
Visual acuity (Snellen denominator)	25.00 (5.13) ^a^	40.63 (9.55) ^b^
Minimum Mini-Mental State Test score		26

Notes: The visual acuity scores for the young in Experiment 2 are based on 20 participants. Standard deviations are in parentheses. Values in the same row that do not share subscripts differ at *p* < 0.05.

#### 2.1.2. Materials

The experimental base-words were 12 taboo (e.g., *shit*, *bitch*) and 12 neutral (e.g., *note*, *frame*) words matched for mean number of letters and syllables in MacKay *et al.* [2]. Using 1–5 scales, naïve participants in MacKay *et al.* rated the taboo words reliably higher than the neutral words for obscenity (*M* = 3.1 *vs.* 1.0) but not for familiarity (*M* = 4.9 and 4.9). Twenty-four additional words (12 taboo and 12 neutral) served as practice stimuli. All words appeared in 48-point font against the white background of a computer monitor.

Following the color-naming and color-recognition tasks, the participants saw the 24 experimental words and rated each word on three dimensions: valence (1 = very negative; 7 = very positive), arousal (1 = not at all calming; 7 = very calming), and familiarity (1 = unfamiliar; 7 = very familiar). The current sample of young adults provided the following scores for the neutral words, *M_valence_* = 4.56 (*SD* = 0.47), *M_arousal_* = 3.46 (*SD* = 0.48), *M_familiarity_* = 6.39 (*SD* = 0.23), and for the taboo words, *M_valence_* = 2.27 (*SD* = 0.68), *M_arousal_* = 5.86 (*SD* = 0.49), *M_familiarity_* = 5.49 (*SD* = 0.52). The current sample of older adults provided the following rating scores for the neutral words, *M_valence_* = 5.61 (*SD* = 0.45), *M_arousal_* = 2.92 (*SD* = 0.48), *M_familiarity_* = 6.86 (*SD* = 0.09), and for the taboo words, *M_valence_* = 2.75 (*SD* = 0.75), *M_arousal_* = 5.64 (*SD* = 0.58), *M_familiarity_* = 6.15 (*SD* = 0.20).

#### 2.1.3. Procedure

Experiment 1 proceeded in four phases: practice, color naming, surprise color-recognition test, and post-experimental ratings for valence, arousal, and familiarity. In the practice phase, participants studied the six color names (blue, brown, gray, green, pink, and red) in their corresponding font colors, and then named the colors of the 24 practice words, receiving experimenter feedback following incorrect responses. 

In the color-naming phase, participants named the font color of the 24 experimental words as quickly as possible while ignoring word meaning. On each trial, a 700 ms fixation point preceded presentation of the word and a 1200 ms blank screen followed the participant’s color-naming response, a relatively brief inter-trial interval intended to discourage further encoding or rehearsal of the stimuli between trials. 

Each of the 24 base-words was repeated six times for a total of 144 color-naming trials, with each participant seeing half the words (six taboo and six neutral) six times in the same font color (the *color-consistent* condition), and half in a different color each time (the *color-inconsistent* condition). Words assigned to the color-consistent *versus* color-inconsistent conditions were counterbalanced across participants and all six colors appeared in each condition for each participant across the 144 trials. 

PsyScope software [21] recorded color-naming RTs via voice key circuitry and presented the stimuli in a pseudorandom order that excluded immediate repetitions of the same word or the same color. The experimenter recorded errors on each trial without providing feedback, and asked participants after the last trial if they noticed that some of the words always appeared in the same color. 

Phase three was a surprise recognition memory test for the color of the 12 color-consistent base-words. On each recognition memory trial, participants saw one of the color-consistent words repeated in a horizontal line across the computer screen six times in one of the six different font colors. Participants then named as quickly as possible the font color that the word assumed in the color-naming phase, guessing if necessary, and indicated confidence in their color-recognition response on a 1–5 scale.

In phase four, participants rated the 24 base-words in the color-naming phase for familiarity, valence, and arousal using 1–7 scales. 

### 2.2. Main Results

Alpha was set at 0.05 for all analyses in Experiments 1 and 2.

#### 2.2.1. Color-Naming Errors

We analyzed color-naming errors (% per condition) via a 2 (age: young *vs.* older) by 2 (word type: neutral *vs.* taboo) by 2 (consistency: color-consistent *vs.* inconsistent) by 2 (repetition: repetitions 1–3 *vs.* 4–6) analysis of variance (ANOVA). There were fewer errors for neutral (*M* = 2.1%, *SD* = 1.1%) than taboo words (*M* = 2.8%, *SD* = 1.5%), *F*(1, 78) = 5.40, *MSE* = .001, *p* = .02, η_p_^2^ = 0.06, and fewer errors for repetitions 4–6 (*M* = 1.9%) than 1–3 (*M* = 3.1%), *F*(1, 78) = 13.20, *MSE* = .002, *p* < 0.001, η_p_^2^ = 0.14. The only significant interaction involved word type and consistency: There were fewer errors for color-inconsistent (*M* = 1.9%) than color-consistent (*M* = 2.3%) *neutral* words, but fewer errors for color-consistent (*M* = 2.3%) than color-inconsistent (*M* = 3.3%) *taboo* words, *F*(1, 78) = 7.09, *MSE* = 0.001, *p* = 0.009, η_p_^2^ = 0.08. No effects involving age were significant.

#### 2.2.2. Color-Naming RTs

Analyses excluded all trials with color-naming errors, microphone errors, and RTs less than 250 ms or greater than 2500 ms (*M* = 3.56% and 3.96% of total trials for young and older adults, respectively). 

**Figure 2:**
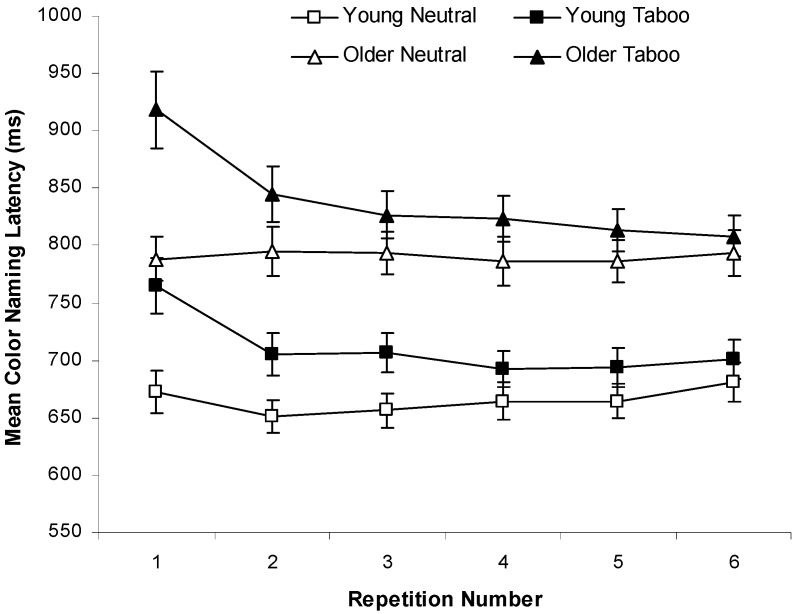
Mean color-naming response times (RTs) (±*SE*) for repetitions 1–6 of neutral *vs.* taboo words for young adults and older adults in Experiment 1.

Figure 2 shows mean color-naming RTs as a function of word type and repetitions 1–6 for young and older adults. RTs did not vary as a function of repetition for neutral words, but decreased between repetition 1 and 2 for taboo words, *F*(1, 79) = 43.40, *MSE* = 16435.62, *p* < 0.001, η_p_^2^ = 0.35, with no reliable RT decreases for subsequent repetitions (see Figure 2). To simplify our figures and the analyses involving *repetition*, we adopted the conservative procedure of grouping repetitions 1–3 *vs.* 4–6. This grouping of repetitions had relatively little effect on the overall pattern of results (*i.e.*, it did not affect the interactions with the *repetition* variable), but provided several advantages, such as simplifying the interpretation of simple effects and limiting the number of tests for simple effects needed to understand interactions.

**Figure 3:**
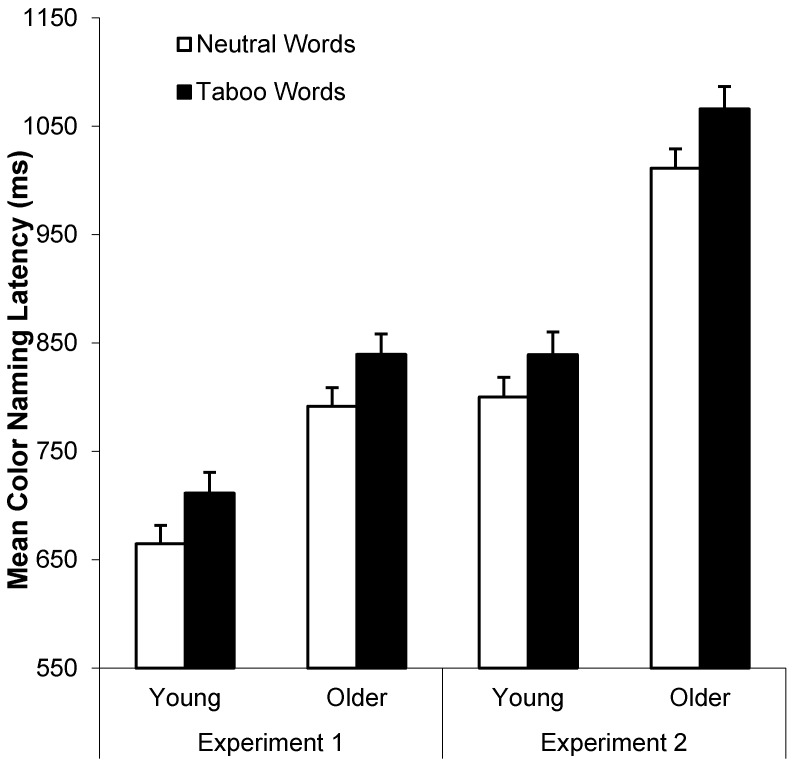
Mean color-naming RTs (+*SE*) as a function of age and word type in Experiments 1 (left panel) and 2 (right panel).

Both young and older adults exhibited taboo Stroop interference (*i.e.*, longer color-naming RTs for taboo than neutral words), as can be seen in Figure 3 (left panel). A 2 (age: young *vs.* older) by 2 (word type: neutral *vs.* taboo) by 2 (consistency: color-consistent *vs.* inconsistent) by 2 (repetition: repetitions 1–3 *vs.* 4–6) ANOVA on these data indicated four main effects: faster color-naming RTs for neutral (*M* = 728 ms, *SD* = 112 ms) than taboo words (*M* = 775 ms, *SD* = 126 ms), *F*(1, 78) = 147.56, *MSE* = 2430.80, *p* < .001, η_p_^2^ = 0.65, faster color-naming RTs for young (*M* = 688 ms, *SD* = 108 ms) than older adults (*M* = 816 ms, *SD* = 130 ms), *F*(1, 78) = 25.69, *MSE* = 101145.10, *p* < 0.001, η_p_^2^ = 0.25, faster color-naming RTs for color-consistent (*M* = 746 ms, *SD* = 116 ms) than color-inconsistent words (*M* = 758 ms, *SD* = 121 ms), *F*(1, 78) = 12.45, *MSE* = 1759.97, *p* = 0.001, η_p_^2^ = 0.14, and faster color-naming RTs for repetitions 4–6 (*M* = 743 ms, *SD* = 111 ms) than repetitions 1–3 (*M* = 761 ms, *SD* = 127 ms), *F*(1, 78) = 10.15, *MSE* = 5320.40, *p* = .002, η_p_^2^ = 0.11.

**Figure 4:**
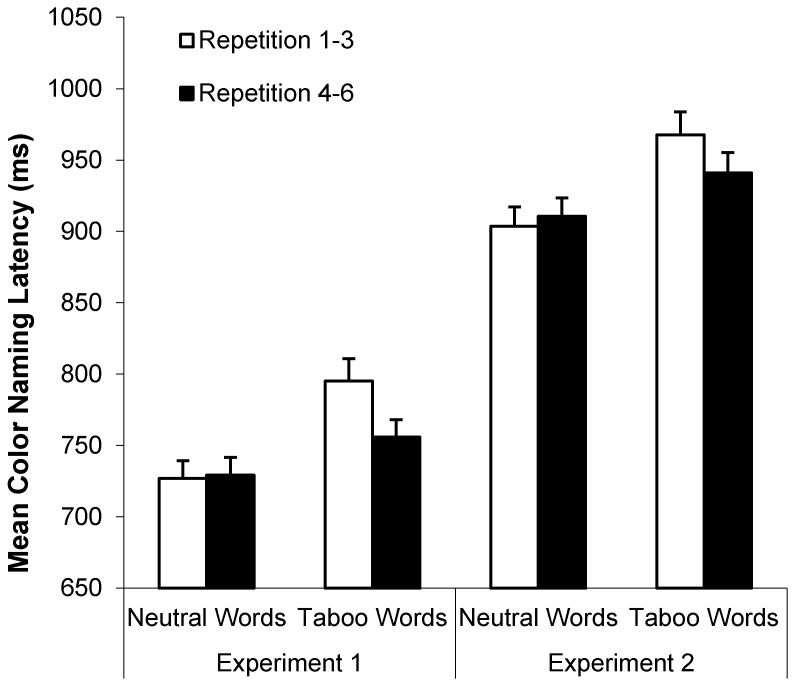
Mean color-naming RTs (+*SE*) as a function of word type and repetitions 1–3 *vs.* 4–6 in Experiments 1 (left panel) and 2 (right panel).

**Figure 5:**
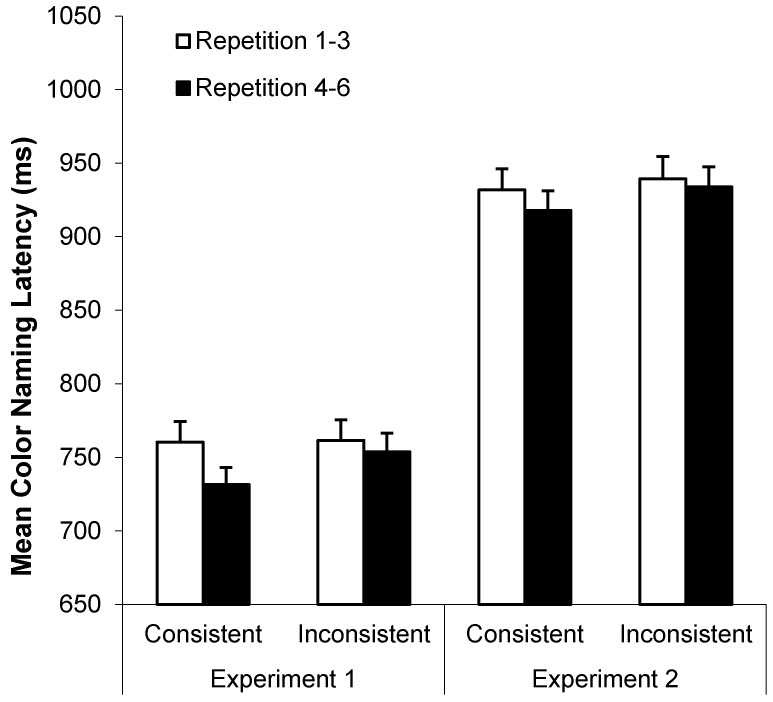
Mean color-naming RTs (+*SE*) as a function of consistency and repetitions 1–3 *vs.* 4–6 in Experiments 1 (left panel) *vs.* 2 (right panel).

**Figure 6:**
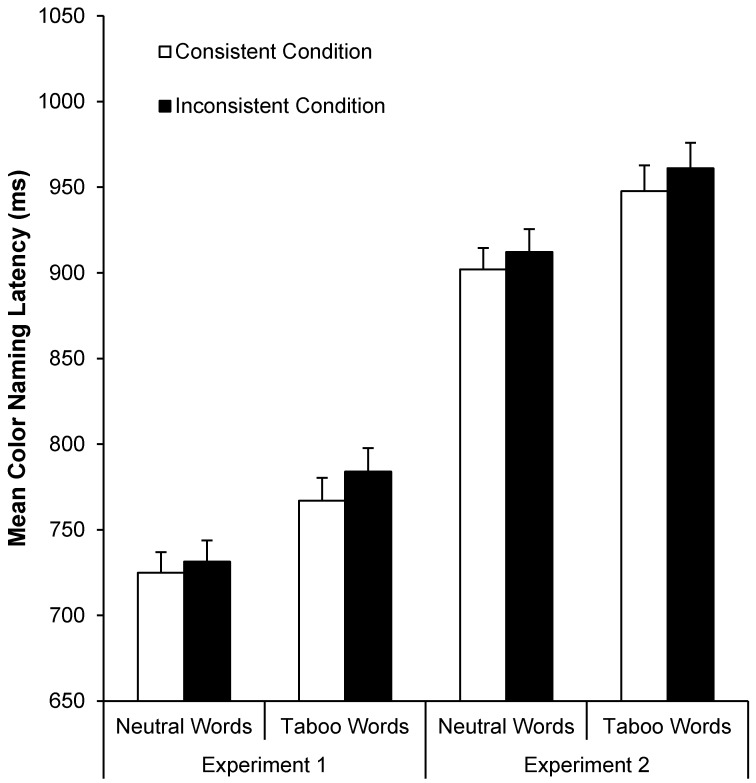
Mean color-naming RTs (+*SE*) as a function of word type and consistency in Experiments 1 (left panel) *vs.* 2 (right panel).

In addition, there were three significant interactions: repetition by word type, *F*(1, 78) = 33.96, *MSE* = 2035.54, *p* < 0.001, η_p_^2^ = 0.30, repetition by color-consistency, *F*(1, 78) = 14.65, *MSE* = 1215.61, *p* < 0.001, η_p_^2^ = 0.17, and word type by color-consistency, *F*(1, 78) = 4.29, *MSE* = 1033.26, *p* = 0.04, η_p_^2^ = 0.05. Figure 4 (left panel) shows the repetition by word type interaction: faster RTs for repetitions 4–6 than 1–3 for taboo words, but not neutral words. Figure 5 (left panel) shows the repetition by color-consistency interaction: a larger RT decrease between repetitions 1–3 and 4–6 for color-consistent than color-inconsistent words. Figure 6 (left panel) and Table 2 (last column) show the interaction between color-consistency and word type: faster RTs for color-consistent than color-inconsistent taboo words, *t*(79) = 3.91, *p* < 0.001, η_p_^2^ = 0.16, but not neutral words. 

Because binding theory and RCT predicted a repetition by consistency interaction for taboo but not neutral words, we explored these *a priori* predictions in planned comparisons using separate ANOVAs for neutral and taboo words. For *taboo* words, RTs were faster for color-consistent than color-inconsistent words, *F*(1, 78) = 15.59, *MSE* = 1477.00, *p* < 0.001, η_p_^2^ = 0.17, for repetitions 4–6 than 1–3, *F*(1, 78) = 23.02, *MSE* = 5327.78, *p* < 0.001, η_p_^2^ = 0.23, and for young than older adults, *F*(1, 78) = 22.82, *MSE* = 57359.92, *p* < 0.001, η_p_^2^ = 0.23. Repetition significantly reduced color-naming RTs for both color-consistent, *t*(79) = 5.57, *p* < 0.001, and color-inconsistent words, *t*(79) = 3.02, *p* = 0.003, but the effect was greater for color-consistent than color-inconsistent words, *F*(1, 78) = 6.89, *MSE* = 1352.31, *p* = 0.01, η_p_^2^ = 0.08 (see Table 2). For *neutral* words, older adults demonstrated the expected slowing effect, and took longer to name the colors than young adults, *F*(1, 78) = 27.91, *MSE* = 46215.99, *p* < 0.001, η_p_^2^ = 0.26. Color-consistency interacted with repetition, *F*(1, 78) = 6.50, *MSE* = 1354.77, *p* = 0.01, η_p_^2^ = 0.08, but importantly for binding theory and RCT, planned comparisons showed that repetition had no significant effect for either color-consistent, *t*(79) < 1.96, or color-inconsistent neutral words, *t*(79) < 1.96 (see Table 2). 

**Table 2:** Mean color-naming RTs for color-consistent *vs.* color-inconsistent words by word type and repetition in Experiment 1.

Word Type	Repetitions		Difference	All Repetitions
	1–3	4–6		
Neutral				
Color-Consistent	729 (13)	720 (12)	−9	725 (12)
Color-Inconsistent	725 (13)	738 (13)	+13	731 (12)
Taboo				
Color-Consistent	792 (16)	742 (12)	−50	767 (13)
Color-Inconsistent	798 (16)	770 (13)	−28	784 (13)

Note: Standard errors are in parentheses.

#### 2.2.3. Recognition Memory for Color

**Figure 7:**
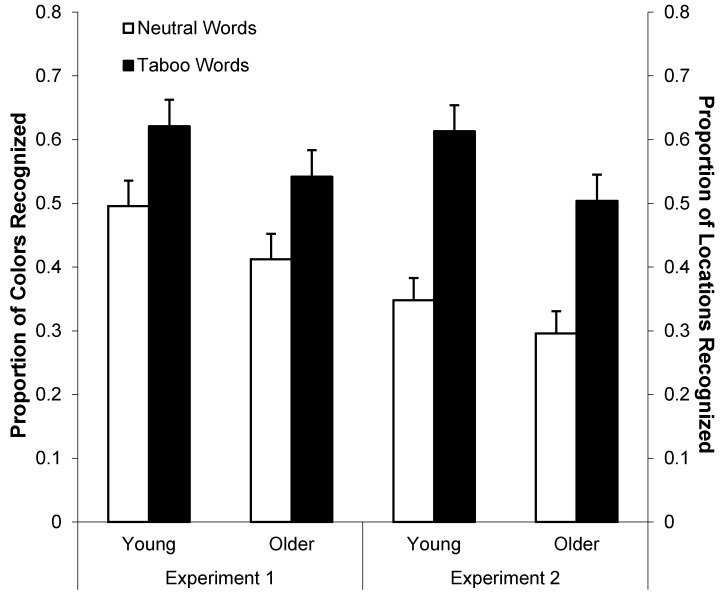
Mean proportion correct recognition memory (+*SE*) for consistent *vs.* inconsistent font colors (Experiment 1, left panel) and consistent *vs.* inconsistent locations (Experiment 2, right panel) as a function of age and word type.

Figure 7 (left panel) shows mean proportion correct color recognition as a function of word type and age group. A 2 (age: young *vs.* older) by 2 (word type: neutral *vs.* taboo) ANOVA on these data indicated taboo context-memory enhancement in both young and older adults: better color recognition for taboo (*M* = 0.58, *SD* = 0.26) than neutral words (*M* = 0.45, *SD* = 0.25), *F*(1, 78) = 17.07, *MSE* = 0.04, *p* < 0.001, η_p_^2^ = 0.18. In addition, color recognition was marginally better for young (*M* = 0.56, *SD* = 0.27) than older adults (*M* = 0.48, *SD* = 0.24), *F*(1, 78) = 2.79, *MSE* = 0.09, *p* < 0.10, η_p_^2^ = 0.03, with no age by word type interaction. 

We analyzed confidence ratings for color recognition decisions in a 2 (age: young *vs.* older) by 2 (word type: neutral *vs.* taboo) by 2 (recognition accuracy: correct *vs.* incorrect) ANOVA. Using this factorial design, 16 participants with either all correct or all incorrect responses for either the neutral or taboo words could not be included in the analysis [22]. Confidence was higher for correct (*M* = 3.35, *SD* = 1.01) than incorrect responses (*M* = 2.20, *SD* = 0.81), *F*(1, 62) = 101.96, *MSE* = 0.83, *p* < .001, η_p_^2^ = 0.62, and higher for taboo (*M* = 3.03, *SD* = 0.82) than neutral words (*M* = 2.52, *SD* = 1.0), *F*(1, 62) = 28.48, *MSE* = 0.59, *p* < 0.001, η_p_^2^ = 0.31. The word type by recognition accuracy interaction was non-significant for the confidence ratings, *p* = 0.79, as were all effects involving age. 

### 2.3. Subsidiary Results

#### 2.3.1. Post-Experimental Ratings for Valence, Arousal, and Familiarity

The supplemental materials report analyses of the post-experimental ratings of word valence, arousal, and familiarity for Experiments 1 and 2. As discussed in detail there, minor age differences emerged in perceived arousal and familiarity that neither qualified nor explained the main results in Experiments 1 and 2 because they did not correlate reliably with size of the taboo Stroop effect. 

#### 2.3.2. Awareness Results

Exactly 62.5% of young and 62.5% of older participants reported awareness of color-consistency during color naming in Experiment 1. An ANOVA on color-naming RTs with awareness, age, and word type as variables yielded the same main effects of age and word type as before but no effect or interaction involving awareness. Despite greater color-recognition memory for aware (*M* = 0.64, *SD* = 0.19) than unaware (*M* = 0.49, *SD* = 0.23) participants, *F*(1, 76) = 6.17, *MSE* = 0.09, *p* = 0.01, η_p_^2^ = 0.08, a similar 2 by 2 by 2 ANOVA on correct color-recognition responses yielded the same main effects of age and word type as before, with no reliable interactions involving awareness. In short, self-reported awareness played no role in any of our effects, including taboo Stroop interference and taboo context-memory enhancement.

#### 2.3.3. Acuity and Vocabulary

As expected, young and older adults differed in visual acuity and vocabulary. To ensure that these differences were not responsible for our observed effects, we ran a series of bivariate correlations between acuity, vocabulary scores, the overall taboo Stroop effect, and the taboo Stroop effects within each combination of the color-consistency and repetition variables. This was done separately by age groups, and for both groups combined. None of the correlations were significant (see the supplemental materials). 

### 2.4. Discussion

Experiment 1 found both of the main effects predicted under RCT and binding theory for young and older adults: taboo Stroop interference (longer color-naming RTs for taboo than neutral words) and taboo context-memory enhancement (superior accuracy and confidence in recognition memory for the font color of taboo *vs.* neutral words in the color-consistent condition). Also consistent with predictions of binding theory and RCT, repetition of words in a consistent font color greatly speeded the color-naming RTs for taboo but not neutral words. 

Because this sharp speed-up in color-naming RTs with repetition of taboo but not neutral words only occurred in the color-consistent condition, it cannot be attributed solely to habituation of emotional reactions to the taboo words. This is because emotional responses would habituate at the same gradual rate with repetition of taboo words regardless of color-consistency.

Turning to aging effects, color-naming RTs were slower and recognition memory was less accurate for older than young adults, but no age differences emerged in taboo Stroop interference or taboo context-memory enhancement. Binding theory and the TDH predicted both of these age-invariant effects. Under binding theory, frequently used units and processes remain intact with aging, and priority context-binding processes are highly practiced, and so are the links between taboo words and the emotional reaction system. 

The present age-invariant results comport with findings in many other studies, e.g., the age-constant speed-up when perceiving threatening *versus* non-threatening faces [23,24] and high *vs.* low arousal pictures [25], and the equivalent Stroop effects for young [26] and older adults [27] processing negative-emotion base-words.

## 3. Experiment 2: Aging and Effects of Location Consistency

Experiment 2 was designed to test the contrasting predictions of RCT *versus* binding theory for location consistency. Procedures in Experiments 1 and 2 were identical except that all words in Experiment 2 were color-inconsistent and location consistency varied: Young and older adults named the font color of words that were either location-consistent (with words recurring six times in the same screen location throughout the task) or location-inconsistent (with words recurring six times in a different screen location each time). They then received a surprise recognition memory test for the screen location of location-consistent words in the color-naming task. 

Both binding theory and RCT predicted slower color-naming RTs for taboo than neutral words, and superior accuracy and confidence in recognition memory for the screen location of taboo compared to neutral words. RCT also predicted a greater speed-up in color-naming RTs with repetition of location-consistent than location-inconsistent taboo words—the same pattern as for color-consistent and color-inconsistent taboo words in Experiment 1. However, binding theory predicted no greater speed-up in RTs with repetition of location-consistent *versus* location-inconsistent taboo words. The reason for this contrast with Experiment 1 is that location-context binding (unlike color-context binding) does not interfere with color naming because different underlying units represent screen location, font color, and selection of the color-naming response in binding theory (see Figure 1).

Concerning effects of aging, binding theory predicted better recognition memory for screen location and higher confidence in screen location responses for young than older adults in Experiment 2, but no effects of aging on color-naming interference or on taboo context-memory enhancement for screen location.

### 3.1. Methods

#### 3.1.1. Participants

Participants were 40 young and 40 older adults resembling those in Experiment 1 (see the background characteristics and statistics in Table 1). All participants gave informed consent for inclusion before participating in the study. The study protocol was approved by the Institutional Review Board at Pomona College.

#### 3.1.2. Materials

The materials were 12 taboo and 12 neutral words from MacKay and Ahmetzanov [4] that were matched for number of syllables and length in letters (see the supplemental materials). Naïve participants using 1–5 scales in MacKay and Ahmetzanov rated the taboo words higher than the neutral words for obscenity (*M* = 3.32 *vs.* 1.03) but not familiarity (*M* = 4.75 *vs.* 4.77). In post-experiment ratings using 1–7 scales with instructions identical to Experiment 1, the current sample of young adults provided the following scores for the neutral words, *M_valence_* = 4.47 (*SD* = 0.32), *M_arousal_* = 3.67 (*SD* = 0.60), *M_familiarity_* = 6.92 (*SD* = 0.10), and for the taboo words, *M_valence_* = 2.39 (*SD* = 0.64), *M_arousal_* = 5.44 (*SD* = 0.52), *M_familiarity_* = 6.70 (*SD* = 0.34). The current sample of older adults provided the following rating scores for the neutral words, *M_valence_* = 6.01 (*SD* = 0.28), *M_arousal_* = 2.75 (*SD* = 0.77), *M_familiarity_* = 6.83 (*SD* = 0.19), and for the taboo words, *M_valence_* = 2.43 (*SD* = 0.51), *M_arousal_* = 5.75 (*SD* = 0.40), *M_familiarity_* = 6.24 (*SD* = 0.31). As the supplemental materials discuss in detail, analyses of the post-experimental valence, arousal, and familiarity ratings for the words in Experiment 2 did not complicate our conclusions. 

#### 3.1.3. Procedures

Each word was presented once in each of six different colors (blue, gray, green, orange, pink, and red), for a total of 144 trials. The words appeared in the six cells in an invisible 2 by 3 spatial grid on the computer screen. Half the words of each type were *location-consistent* and always appeared in the same cell location, while the other half were *location-inconsistent* and appeared only once in each cell location. The specific words in the location-consistent *versus* location-inconsistent conditions were counterbalanced between participants across two versions of the experiment. 

Procedures were identical to phases 1–4 in Experiment 1 except that the fixation point preceding stimulus presentation in the color-naming phase was 500 ms, and to allow error coding, the screen went blank after each color-naming response (*M* = 877 ms) and remained blank for an additional 1000 ms before the next trial. As before, words were presented in a pseudo-random order such that adjacent stimuli never had identical words, colors, or locations. 

Phase 3 was a surprise recognition memory test for the screen location of the 12 location-consistent words in the color-naming phase. On each recognition-memory trial, participants saw one of the location-consistent words repeated in all six locations in the invisible grid, and identified its original screen location either verbally (e.g., “upper left”) or by pointing. Participants were encouraged to respond as quickly as possible, and to guess if necessary. They then rated confidence in their location memory decision on a 1–5 scale. 

### 3.2. Main Results

#### 3.2.1. Color-Naming Errors

As in Experiment 1, we analyzed color-naming errors (percentage of trials) in a 2 (age: young *vs.* older) by 2 (word type: neutral *vs.* taboo) by 2 (consistency: location-consistent *vs.* inconsistent) by 2 (repetition: repetitions 1–3 *vs.* 4–6) ANOVA. Fewer errors occurred for location-consistent (*M* = 1.9%, *SD* = 3.3%) than location-inconsistent (*M* = 2.6%, *SD* = 3.8%) words, *F* (1, 78) = 7.34, *MSE* = .001, *p* = 0.008, η_p_^2^ = 0.09, and young adults made fewer errors (*M* = 1.7%, *SD* = 2.9%) than older adults (*M* = 2.8%, *SD* = 4.0%), *F* (1, 78) = 7.55, *MSE* = 0.002, *p* = 0.007, η_p_^2^ = 0.09. The only other effect to approach significance was a marginal interaction between age and word type, *F*(1, 78) = 3.33, *MSE* = 0.002, *p* = 0.072, η_p_^2^ = 0.04: Older adults made more errors on taboo (*M* = 3.3%, *SD* = 4.7%) than neutral words (*M* = 2.2%, *SD* = 3.3%), but error rates for the young were similar across word types (*M* = 1.8%, *SD* = 3.1% and 1.7%, *SD* = 2.9% of taboo and neutral words, respectively).

#### 3.2.2. Color-Naming RTs

Trials with color-naming errors, microphone errors, or RTs less than 250 ms or greater than 2500 ms were excluded in all RT analyses (*M* = 5.0% and 6.4% of all trials for young and older adults, respectively). Figure 3 (right panel) shows mean color-naming RTs as a function of word type and age group. A 2 (age: young *vs.* older) by 2 (word type: neutral *vs.* taboo) by 2 (consistency: location-consistent *vs.* location-inconsistent) by 2 (repetition: repetitions 1–3 *vs.* 4–6) ANOVA on these data indicated faster color-naming RTs for young (*M* = 822 ms, *SD* = 100 ms) than older adults (*M* = 1038 ms, *SD* = 154 ms), *F*(1, 78) = 63.85, *MSE* = 116768.78, *p* < 0.001, η_p_^2^ = 0.45, faster color-naming RTs for neutral (*M* = 907 ms, *SD* = 119 ms) than taboo words (*M* = 954 ms, *SD* = 136 ms), *F*(1, 78) = 81.07, *MSE* = 4401.46, *p* < 0.001, η_p_^2^ = 0.51, and faster color-naming RTs for location-consistent (*M* = 924 ms, *SD* = 125 ms) than location-inconsistent (*M* = 936 ms, *SD* = 130 ms) words, *F*(1, 78) = 13.43, *MSE* = 1647.13, *p* < 0.001, η_p_^2^ = 0.15. Only the repetition by word type interaction was significant, *F*(1, 78) = 26.90, *MSE* = 1679.90, *p* < 0.001, η_p_^2^ = 0.26, with longer naming RTs for repetitions 1–3 than 4–6 for taboo but not neutral words (see Figure 4, right panel).

**Figure 8:**
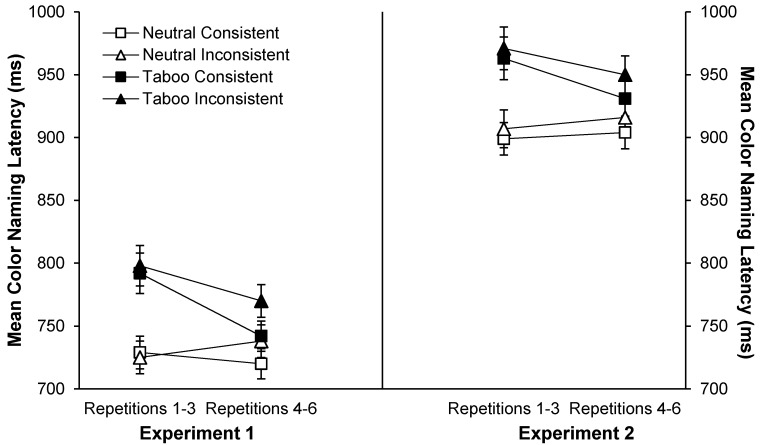
Mean color-naming RTs (±*SE*) as a function of consistency and word type in Experiments 1 (left panel) and 2 (right panel).

In contrast to the color-consistency results in Experiment 1, no interactions involving location-consistency were significant (see Figure 5 and Figure 6, right panels). To render our planned comparisons parallel to Experiment 1, we conducted separate age by consistency by repetition ANOVAs for both word types. Importantly for binding theory, repetition of location-consistent words did not reliably speed up color-naming time in Experiment 2, unlike the repetition of color-consistent words in Experiment 1. Comparing Table 3 (Experiment 2) to Table 2 (Experiment 1), the differences in color-naming RTs between repetitions 1–3 and 4–6 for each word type and consistency condition were similar across experiments with one critical exception: taboo words in the location- *vs.* color-consistent conditions (see Figure 8). The speeding up of RTs with repetition did not differ significantly between location-consistent and location-inconsistent taboo words in Experiment 2, but in Experiment 1, the speeding up with repetition for color-consistent taboo words was almost double that for color-inconsistent taboo words.

**Table 3:** Mean color-naming RTs for location-consistent *vs.* location-inconsistent words by word type and repetition in Experiment 2.

Word Type	Repetitions		Difference	All Repetitions
	1–3	4–6		
Neutral				
Location-Consistent	899 (13)	904 (13)	+5	902 (12)
Location-Inconsistent	907 (15)	916 (13)	+9	912 (13)
Taboo				
Location-Consistent	963 (17)	931 (15)	−32	947 (15)
Location-Inconsistent	971 (17)	950 (15)	−21	961 (15)

Note: Standard errors are in parentheses.

#### 3.2.3. Recognition Memory for Location

Figure 7 (right panel) shows mean correct location recognition proportions as a function of age and word type. A 2 (age: young *vs.* older) by 2 (word type: neutral *vs.* taboo) ANOVA on these data indicated a main effect of word type, *F*(1, 78) = 47.05, *MSE* = 0.05, *p* < 0.001, η_p_^2^ = 0.38, with better location-recognition for taboo (*M* = 0.56, *SD* = 0.26) than neutral words (*M* = 0.32, *SD* = 0.22). There was no main effect or interaction involving age, indicating taboo location-memory enhancement for both young and older adults. 

We analyzed confidence ratings for location-recognition decisions in a 2 (age: young *vs.* older) by 2 (word type: neutral *vs.* taboo) by 2 (recognition accuracy: correct *vs.* incorrect) ANOVA. This mixed factorial design required dropping 21 participants (8 young, 13 older) who had either all correct or all incorrect responses in any cell. Confidence ratings were higher for correct (*M* = 2.94, *SD* = 1.21) than incorrect (*M* = 2.24, *SD* = .90) responses, *F*(1, 57) = 52.71, *MSE* = 0.55, *p* < 0.001, η_p_^2^ = 0.48, and for taboo (*M* = 2.87, *SD* = 1.09) than neutral (*M* = 2.30, *SD* = 1.02) words, *F*(1, 57) = 37.75, *MSE* = 0.50, *p* < 0.001, η_p_^2^ = 0.40. The only significant interaction involved age and recognition accuracy, *F* (1, 57) = 11.39, *MSE* = 0.55, *p* = 0.001, η_p_^2^ = 0.17. For correct responses, young were more confident than older adults, but for incorrect responses, older adults were more confident than young adults, with mean confidence ratings in incorrect responses falling below the midpoint of the scale for both age groups.

### 3.3. Subsidiary Results

#### 3.3.1. Awareness Results

Awareness of location-consistency during color naming was reported by 57.5% of young and 32.5% of older participants. An ANOVA on color-naming RTs with awareness, age, and word type as variables again yielded main effects of age and word type, but no effect or interaction involving awareness. A similar 2 by 2 by 2 ANOVA on proportion of correct location-recognition responses yielded marginally better location-recognition for aware (*M* = 0.50, *SD* = 0.23) than unaware participants (*M* = 0.42, *SD* = 0.24), *F*(1, 76) = 3.50, *MSE* = .06, *p* = 0.07, η_p_^2^ = 0.04. Awareness also interacted with age, *F*(1, 76) = 10.66, *MSE* = 0.06, *p* = 0.002, η_p_^2^ = 0.12: For the young there was no significant difference in recognition memory between aware and unaware participants, but older aware participants recognized a larger proportion of locations (*M* = 0.54, *SD* = 0.24) than the older unaware (*M* = 0.33, *SD* = 0.22), *t*(38) = 3.56, *p* = 0.001. The interaction between awareness and word type was non-significant, indicating that awareness affected neither taboo Stroop interference nor taboo location-memory enhancement. 

#### 3.3.2. Acuity and Vocabulary

As in Experiment 1, there were age differences in acuity and vocabulary, which we examined in the same manner as in Experiment 1, but again, none of the correlations with taboo Stroop effects was significant (see the supplemental materials). 

#### 3.3.3. Post-experimental Ratings for Valence, Arousal, and Familiarity

The supplemental materials present detailed analyses of the post-experiment ratings for valence, arousal, and familiarity. As in Experiment 1, age differences in these ratings did not qualify the current results.

### 3.4. Discussion

Experiment 2 yielded the three taboo Stroop effects predicted under binding theory and the RCT for young and older adults: longer color-naming RTs for taboo than neutral words (taboo Stroop interference) and superior accuracy and confidence in incidental recognition memory for the location of taboo compared to neutral words (taboo context-memory enhancement). Also consistent with binding theory and RCT, color-naming RTs remained constant with repetition of neutral words but decreased gradually with repetition of taboo words—a finding readily explained by habituation of the emotional reactions to taboo words. 

Turning to unique theoretical predictions for Experiment 2, RCT predicted a greater decrease in color-naming RTs with repetition of location-consistent than location-inconsistent taboo words, an interaction that was not observed. Unlike the color-consistency effect in Experiment 1, naming RTs decreased to a similar extent with repetition of location-consistent and location-inconsistent taboo words. The contrasting effects of color- *vs.* location-consistency were not predicted by RCT: If emotion-linked images underlie the speed-up in color-naming RTs with repetition of taboo words, repetition effects should not differ for color *vs.* location because font color and screen location are simultaneously present in the image for a word. 

Binding theory, however, predicted these differing effects for color- *vs.* location-consistency. Under binding theory, the binding of a taboo word’s lexical node to its font color is prioritized, and interferes with the binding of font color to the color name response (see Figure 1). With repetition of color-word combinations, repeated binding of the lexical node to the font color need not occur, thus eliminating the source of interference. However, the process that binds a taboo word’s lexical node to its screen location is independent of the process that binds font color to the color-naming response. Priority binding of the lexical node to the screen location does not interfere with the binding of font color to the color name response, and repeating a particular location-word combination does not eliminate the source of the interference.

Of course, a significant experiment x consistency x repetition interaction for the taboo words would have strengthened these conclusions. However, a reliable three-way interaction is technically unnecessary given our a priori theoretical prediction that results for the two experiments would differ and given the following simple explanation for why the interaction was non-reliable: On average, RTs were reliably slower in Experiment 2 than Experiment 1, an almost 200ms difference that quite likely reflects the eye movements necessary in Experiment 2 to focus on the unpredictable screen locations of the stimuli. No such eye movements were necessary in Experiment 1 because the stimuli always appeared at the central focus point. This 200ms difference would make a small but real between-experiment interaction extremely difficult to detect. To strengthen present conclusions, the challenge for future studies will be to find a methodology that minimizes overall difference in response times in the color- *versus* location-consistent conditions. 

Turning to aging, taboo Stroop interference and taboo context-memory enhancement were age-invariant, replicating the age-invariance observed in Experiment 1 and other studies [23,24,25,26,27]. Binding theory predicted this age-invariance because both taboo Stroop interference and context-memory enhancement involve frequently used units and processes that remain intact with aging under the TDH: taboo words with frequently used semantic representations and connections to the emotional reaction system, and highly practiced priority binding processes.

## 4. General Discussion

### 4.1. Other Theories of Aging, Memory, and Emotion

Present results bear on four additional aging theories discussed next: inhibition deficit theory, RCT, socioemotional selectivity theory, and the associative deficit hypothesis. 

#### 4.1.1. Inhibition Deficit Theory

Over a hundred years ago, Pavlov suggested that inhibitory processes are especially vulnerable to effects of aging, and the inhibitory theory of attention and aging known as the inhibition deficit theory (IDT) makes a similar assumption (e.g., [28,29]). Under IDT, the processing of task-irrelevant information is more difficult to inhibit for older than young adults, causing age-linked increases in recall of and interference from task-irrelevant information (e.g., [30,31,32,33,34]; but see [35], for alternative interpretations). 

Turning to emotion under IDT, Wurm, LaBouvie-Vief, Aycock, Rebucal, and Koch [36] argued that task-irrelevant stimuli that are emotionally arousing are especially difficult for older adults to inhibit. Their primary data consisted of auditory lexical decision times for “emotion words” (e.g., *fear*) spoken in an emotional tone that was either congruent or incongruent with the emotion that the word designates. The results indicated that incongruent word-voice tone stimuli slowed RTs for older but not young participants, as if older adults are less able to ignore or inhibit the emotion-linked voice quality. Wurm *et al.* also observed slower RTs to name the font colors of higher *vs.* lower arousal words in an emotional Stroop task, but only for older adults, not young adults. 

These Wurm *et al.* [36] results contrast with a wide range of age-invariant findings. First, contrary to Wurm *et al.*, Experiment 1 and other studies found equivalent effects of strong emotional arousal on cognitive processing in young and older adults, for example, age-invariant speed-up when detecting threatening *vs.* non-threatening faces [23,24] and when identifying high *vs.* low arousal pictures [25]. Second, in previous emotional Stroop studies, young adults have shown greater Stroop interference for high than low arousal base-words (e.g., [26,37]), whereas arousal effects were absent for young adults in Wurm *et al.* Third, previous studies revealed reliable Stroop interference for negative- but not positive-emotion words in young [26] and older adults [27], whereas Wurm *et al.* reported no overall effect of emotional valence on Stroop interference. 

Further tests of the specific emotion-linked inhibitory processes postulated in IDT are therefore needed, and the taboo Stroop task provides a wonderful context for doing so because Stroop interference is widely believed to reflect the efficiency of inhibitory processes (e.g., [38,39,40,41]). If task-irrelevant emotion-linked word meanings are especially difficult to inhibit (as [36] suggests), then the age-linked inhibition deficits assumed in IDT predict greater processing of task-irrelevant taboo word meanings for older adults in the taboo Stroop task, and thus a larger increase in color-naming RTs for taboo relative to neutral words for older than young adults. IDT also predicts relatively better memory for the font color of taboo than neutral words for older relative to young adults because enhanced processing of taboo word meanings should increase the likelihood that older adults will encode these meanings in relation to their context. Although failure to find reliable effects does not prove their non-existence, Experiment 1 results challenge IDT because none of these predicted age effects was observed. 

With the same caveat, Experiment 2 results also challenge IDT, which predicts relatively better memory for the screen location of taboo than neutral words for older relative to young adults and relatively greater color-naming interference for older relative to young adults. We observed neither of these predicted age effects, contrary to the hypothesized age-linked impairment of inhibition for task-irrelevant word meanings (e.g., [28]) and task-irrelevant emotional reactions in Stroop tasks [36].

#### 4.1.2. Socio-Emotional Selectivity Theory and RCT Capacity Limitations

Under socio-emotional selectivity theory (SST), emotional gratification becomes increasingly important to older adults as they approach the end of their lifespan, an evaluative change that motivates them to devote more attentional resources to reducing the impact of negative emotional information (e.g., [42,43,44,45,46]). Moreover, because SST adopts the RCT assumption that attentional capacity is limited, with less overall capacity for older than young adults, older adults will devote relatively more attentional resources than young adults to cognitive control processes for ignoring negative-valence emotional stimuli under SST, the end result being a positivity bias (e.g., [47]).

These SST assumptions explain three age-related patterns in the literature. First, despite the increased likelihood of illness, loss of friends, and their own approaching death, older adults report fewer negative emotional experiences than young adults (e.g., [45,48,49]). Second, older adults report better control of their emotions than young adults (e.g., [50]), even though declines with aging in the brain regions implicated in cognitive, emotional and executive control have been well established (e.g., [51,52,53,54,55,56]). Third, older adults attend less than young adults to simultaneously presented faces with negative than neutral expressions [57,58] and show less benefit than young adults in remembering negative pictures [45] and words [59] relative to neutral stimuli. 

Within the Taboo Stroop paradigm, age-linked reductions in overall capacity, together with greater capacity devoted to avoiding negative information, implies reduced capacity in older relative to young adults for naming the font color of taboo words and for encoding and remembering the relation between taboo words and their context. As a consequence, SST predicts relatively greater taboo Stroop interference and relatively poorer memory for the font color and screen location contexts of taboo *versus* neutral words for older than young adults. Because neither age effect was observed, present results are problematic for this standard version of SST.

However, SST also generates an alternate prediction for effects of aging on taboo Stroop interference. If older adults successfully avoid processing negative information (as in [57,58]), they may process taboo word meanings less strongly than young adults, thereby reducing interference when naming the font color of taboo words. This version of SST predicts *less* rather than more taboo Stroop interference for older relative to young adults, and less taboo context-memory enhancement because less attention to taboo word meanings implies less ability to remember their associated contextual features in the color- and location-consistent conditions. Neither age effect was observed in Experiments 1 and 2, raising problems for this alternate version of SST. 

To explain why so many studies have failed to observe the age-related positivity biases predicted under SST (see the recent meta-analysis of [60]), Mather [47] suggested that tests for positivity effects must meet three preconditions: The task must be amenable to socio-emotional goals and control processes, it must not exceed available resources for cognitive control, and it must not constrain cognitive processing (consistent with recent results indicating that when tasks constrain cognitive processes, age differences in emotion-related attentional biases disappear; see [60]). 

As the classic paradigm for demonstrating cognitive control processes, Stroop tasks [40,41,61,62] clearly satisfy some of Mather’s preconditions, and both taboo and *emotional* Stroop tasks meet all three preconditions because they engage precisely the sort of socio-emotional goals and control processes said to support SST. For example, optimists experience greater Stroop interference for positive than negative emotion words, consistent with their optimistic socio-emotional framework, and the opposite holds for pessimists, consistent with their pessimistic socio-emotional framework [63]. Because these considerations suggest that the taboo Stroop task represents an appropriate platform for demonstrating age-linked positivity effects, the present age-invariance for taboo Stroop interference and taboo context-memory enhancement remain problematic for SST. 

#### 4.1.3. Associative-Deficit Hypothesis

Under the Associative-Deficit Hypothesis (ADH; [64]), aging reduces the ability to bind together multiple attributes within a single event as well as pairs of concurrently presented stimuli. Consistent with ADH, associative memory deficits have been demonstrated for word pairs [64], picture pairs [65], names and faces [66], a word and its font, and between events and their contexts of occurrence in explicit episodic memory tasks [64], and two studies suggest that older adults continue to experience associative memory deficits for valenced or arousing stimuli [67,68]. 

Also consistent with the age-related associative memory declines in the ADH, older adults in Experiment 1 were marginally worse than young adults at recognizing the font color of color-consistent words. However, ADH did not predict the present age constancy for effects of emotion. Intra-item binding improved to the same extent for young and older adults processing color-consistent and location-consistent taboo relative to neutral words in Experiments 1 and 2.

## 5. Conclusions

Experiments 1 and 2 demonstrated three reliable and age-invariant effects of emotion in the taboo Stroop task: *taboo Stroop interference* (longer color-naming RTs for taboo than neutral base-words, an effect that gradually diminished with word repetition); *taboo context-memory enhancement* (better incidental recognition memory for font colors and screen locations consistently associated with taboo words compared to neutral words); and *repetition by consistency effects for taboo but not neutral words and for color- but not location- consistency* (greater speed-up in color-naming RTs with repetition of color-consistent than color-inconsistent taboo words, but no difference with repetition of location-consistent *versus* location-inconsistent taboo words). These contrasting effects of repetition on taboo Stroop interference as a function of color- *vs.* location-consistency were predicted under binding theory, but not under RCT. 

An alternate suggestion for explaining the present results is that emotion influences memory and cognition in two ways: by altering attention (as an undefined intuitive process) and by increasing general arousal, thereby facilitating retention (see [69]). However, there is no obvious reason why effects of repetition on attention or arousal would differ for the color- *vs.* location-consistency conditions in the current study.

This two-process (attention or arousal) idea also faces other challenges. For example, in immediate recall of RSVP lists, taboo words are better recalled than neutral words only in mixed taboo-neutral lists: In pure (all-taboo or all-neutral) lists with the same words and the same presentation rates, recall of taboo and neutral words does not differ, contrary to the idea that arousal (like attention) directly strengthens memory for emotional words (see [12]). The contrasting effects of attention and arousal in MacKay and Ahmetzanov [4] are also difficult to explain under the two-process idea: Attention (a well-defined concept in color-naming tasks) *enhanced* location-memory for the font colors in color-consistent locations in MacKay and Ahmetzanov, whereas arousal (taboo *vs.* neutral words) had no effect on location-memory for *colors* appearing in color-consistent locations. 

Turning to aging, effects of emotion observed here were age-constant, a finding consistent with previous results indicating age constancy in the cognitive benefits of accessing negative-valence high-arousal emotional concepts, e.g., faster detection times for threatening *vs.* non-threatening faces [23,24] and for high *vs.* low arousal pictures [25]. Together with previous results, Experiments 1 and 2 therefore suggest that high arousal negative valence concepts have age-invariant effects for words, faces, and pictures. 

It is unlikely that the present age-invariance in effects of emotion reflect deliberate shifts in attention toward emotional concepts because we instructed participants to attend and respond to font color and to ignore the meaning of the words, and we excluded errors in our RT analyses. It is also unlikely that the age-invariance occurred because taboo Stroop performance is impervious to control processes, immune to inhibition of task irrelevant information, or unaffected by attentional biases against negative-emotion stimuli. Also unlikely is the possibility that the present age-invariant results reflect insufficient power to detect differences. Estimating a medium sized effect of η_p_^2^ = 0.10 (which is smaller than most of the effects observed in Experiment 1 and 2), and assuming no more than a minimal correlation between repeated measures (*r* = 0.20), a sample size of N = 80 produced a power estimate of .83. Power was more than adequate to detect the usual age-linked slowing (of color-naming RTs), as well as the common (but not universal) observation that older adults report greater confidence than young adults in their erroneous responses (including erroneous location-recognition responses; see Experiment 2 and [70]). 

Binding theory predicted the present age-invariant effects of emotion because emotionally charged stimuli trigger age-invariant emotional reactions that prioritize binding of the source of emotion to contextual features. The binding theory of emotion and aging therefore warrants further test in the taboo Stroop and other paradigms.

Nonetheless, binding theory and the preservation with aging of emotion-linked interference and context binding effects cannot explain why older adults sometimes show relatively poorer memory than young adults for emotional information with negative but not positive valence and why this pattern emerges under full but not divided attention and for individuals with high but not low cognitive control abilities (see [46]). These phenomena suggest that some older adults in some situations do apply strategic control processes to selectively enhance their memory for positive relative to negative information, consistent with SST. 

Why then did such age-linked control biases not emerge in the present research? The most plausible hypothesis is that our naming RTs were relatively rapid (leaving little time for strategic processes) and our memory tests were unexpected (eliminating incentives for participants to engage in strategic encoding processes). This being the case, the relation between emotion, memory, and aging may depend in part on whether the information processing situation triggers fast-reaction, involuntary [71] binding processes, as in the present research, or whether circumstances allow strategic processes of the sort that SST postulates (see, e.g., [72]). The interplay between aging, fast reaction, involuntary processes, and cognitive control processes clearly represents an important issue for future research on relations between emotion, memory, and attention (see also [56,73]).

## Supplementary Files

Supplementary File 1
