# Supplementary material for: Aging, Emotion, Attention, and Binding in the Taboo Stroop Task: Data and Theories
Source: Int J Environ Res Public Health. 2015 Oct 14;12(10):12803–33. doi: 10.3390/ijerph121012803 (PMC4627001; doi:10.3390/ijerph121012803)
Supplement: Supplementary File 1 [file ijerph-12-12803-s001.pdf]

Supplementary Information

Aging, Emotion, Attention, and Binding in the Taboo Stroop Task: Data and Theories

These supplementary materials report analyses of acuity and post-experimental rating measures in Experiments 1 and 2 of MacKay, Johnson, Graham and Burke [1], where the primary task was to name the font color of taboo and neutral words as quickly as possible (after [2]). Acuity was measured pre-experimentally and participants rated the experimental stimuli for valence, arousal, and familiarity in a post-experimental rating task. The present goal was to determine whether the non-reliable age differences in taboo Stroop interference in MacKay *et al.* [1] reflect differences between young and older adults in acuity and rated valence, arousal, or familiarity. We therefore compared acuity and post-experimental ratings of young and older adults and then correlated these measures with the degree of Stroop interference (defined as the percent difference in mean color naming times for taboo *vs.* neutral words) in Experiments 1 and 2 of MacKay *et al* [1].

1. Experiment 1

1.1. Methods

Participants were 40 young (M = 19.2 years) and 38 older adults (M = 72.0 years). Snellen acuity was better for the young (M = 21.50, SD = 2.82) than older adults (M = 34.75, SD = 9.80), $t(78)$ = 8.22, but this age difference did not complicate the conclusions in MacKay *et al.* [1] because the size of the taboo Stroop interference effect (percent difference in mean color-naming times for taboo *vs.* neutral words) did not correlate reliably with Snellen acuity.

1.2. Rating Procedures

Following the color naming and color recognition tasks in MacKay *et al.* [1], the participants saw the 24 experimental words presented in the color naming task (see Table S1) and rated each word on three dimensions: valence (1 = very negative; 7 = very positive), calmness (1 = not at all calming; 7 = very calming), and familiarity (1 = unfamiliar; 7 = very familiar).

1.3. Results and Discussion

To better match the existing literature on ratings for emotional stimuli, we re-scaled each participant's calmness ratings to reflect "arousal" instead, so that a score of 7 (very calming) became a score of 1 (not at all arousing). Mean ratings for valence, arousal, and familiarity are shown by word and word type for young and older adults in Table S1 (the ratings for two older adults were lost due to experimenter error).

We conducted separate age by word type ANOVAs for each rating dimension. Valence ratings were more positive for neutral (M = 5.08) than taboo words (M = 2.5), $F(1, 76)$ = 498.50, MSE = 0.52, p < 0.001, n_p^2 = 0.87, and more positive for older (M = 4.17) than young adults (M = 3.41), F (1, 76) = 26.36, MSE = 0.86, p < 0.001, n_p^2 = 0.26. Valence ratings also yielded a reliable age by

word type interaction, F (1, 76) = 6.10, MSE = 0.52, p = 0.016, n_p^2 = 0.07, with older adults generating relatively more positive ratings for neutral than taboo words compared to young adults (see Table S1). Arousal ratings were lower for neutral than taboo words (M's = 3.21 and 5.76, respectively), F(1, 76) = 486.14, MSE = 0.52, p < 0.001, n_p^2 = 0.87, and lower for older than young adults (M's = 4.31 and M = 4.66, respectively), F(1, 76) = 7.14, MSE = 0.67, p = 0.009, n_p^2 = 0.09, with no age by word type interaction. Familiarity ratings were higher for neutral (M = 6.62) than taboo words (M = 5.83), F(1, 76) = 43.10, MSE = 0.57, p < .001, n_p^2 = .36, and higher for older (M = 6.52) than young adults (M = 5.93), F(1, 76) = 8.93, MSE = 1.50, p = 0.004, n_p^2 = 0.11, with no familiarity by word type interaction.

Table S1. Base-words in Experiment 1 with length in letters and mean post-experimental ratings by word, word type, rating type, and age.

Word Type	Item	Word Length	Mean Valence Rating		Mean Arousal Rating		Mean Familiarity Rating	
			Young	Older	Young	Older	Young	Older
Neutral	Attic	5	4.20	5.50	3.68	3.04	6.35	6.89
	Bank	4	4.43	5.83	3.78	3.09	6.53	6.95
	Brother	7	5.48	6.23	2.45	2.06	6.83	6.89
	Cross	5	4.33	5.56	3.75	2.80	6.25	6.82
	Frame	5	4.25	5.40	3.55	3.05	6.18	6.82
	Host	4	4.95	6.10	3.15	2.51	6.13	6.92
	Lung	4	4.48	5.49	3.48	3.21	6.43	6.97
	Note	4	4.55	5.64	3.30	2.65	6.65	6.87
	Page	4	4.40	5.43	3.55	3.01	6.55	6.87
	Pity	4	3.85	4.76	4.25	3.79	6.18	6.67
	Senate	6	4.45	5.06	3.83	3.48	6.10	6.73
	Wife	4	5.33	6.27	2.81	2.33	6.44	6.95
Mean		4.67	4.56	5.61	3.46	2.92	6.39	6.86
SD		0.98	0.47	0.45	0.48	0.48	0.23	0.09
Taboo	Anus	4	2.98	4.06	5.65	4.78	5.45	6.35
	Bitch	5	1.80	2.40	6.00	5.97	5.93	6.05
	Cock	4	2.65	2.88	5.83	5.38	5.53	6.14
	Dyke	4	1.83	2.90	5.95	5.47	4.63	5.75
	Nigger	6	1.23	1.74	6.64	6.28	4.88	6.09
	Piss	4	2.7	2.45	5.38	5.76	6.05	6.28
	Pussy	5	2.28	2.91	5.93	5.41	5.46	5.95
	Queer	5	3.08	2.89	5.05	5.40	5.25	6.20
	Rape	4	1.3	2.11	6.78	6.71	5.63	6.48
	Scrotum	7	3.23	4.18	5.48	4.76	4.83	6.29
	Shit	4	2.35	2.48	5.65	5.60	6.23	6.23
	Slut	4	1.75	2.01	5.98	6.15	6.00	6.04
Mean		4.67	2.27	2.75	5.86	5.64	5.49	6.15
SD		0.98	0.68	0.75	0.49	0.58	0.52	0.20

The present effect of valence comports with that for naïve young adults rating identical stimuli using 1–5 scales in MacKay and Ahmetzanov [3], with higher positive valence ratings for neutral than taboo words. However, the present main effect of familiarity conflicted with the non-effect of

familiarity for naïve young adults in MacKay and Ahmetzanov [3], where mean familiarity was identical for taboo and neutral words.

Turning to age differences in the present analyses, we wondered whether the age-invariance in taboo Stroop interference reported in MacKay *et al.* [1] was related to participant-specific perceptions of valence, arousal, and familiarity determined post-experimentally. To explore this issue, we computed bivariate correlations between the degree of taboo Stroop interference (percent difference in mean color naming times for taboo *vs*. neutral words) and the mean valence, arousal, and familiarity ratings by word type for young adults (see Table S2) and older adults (see Table S3). As in MacKay *et al.*, we excluded color-naming trials involving errors, microphone errors, and latencies greater than 2500 ms in calculating mean color naming times and, by extension, taboo Stroop interference effects. For young adults, there was a small negative correlation between valence ratings of taboo words and the degree of taboo Stroop interference: Young participants who exhibited greater taboo Stroop interference rated the taboo words as somewhat more negative. For older adults, there was a positive correlation between familiarity ratings for taboo words and the degree of taboo Stroop interference. However, interpretation of this familiarity correlation was complicated by ceiling effects (18 of the 40 older participants rated all of the taboo words as "7 = highly familiar"), and after removing these 18 participants from the analysis, the correlation was non-significant, $r(20) = 0.39$.

Table S2. Bivariate correlations of taboo Stroop interference, valence, arousal, and familiarity ratings for young adults in Experiment 1.

Variable	1	2	3	4	5	6	7
1. Taboo Stroop Interference	–	0.06	−0.34 *	0.24	−0.27	0.31	0.09
2. Neutral Valence		–	−0.21	0.77 **	−0.26	0.11	0.09
3. Taboo Valence			–	−0.18	0.74 **	−0.29	0.04
4. Neutral Arousal				–	−0.25	0.05	0.17
5. Taboo Arousal					–	−0.31	−0.06
6. Neutral Familiarity						–	0.59 **
7. Taboo Familiarity							–

* $p < 0.05$; ** $p < 0.01$.

Table S3. Bivariate correlations of taboo Stroop interference, valence, arousal, and familiarity ratings for older adults in Experiment 1.

Variable	1	2	3	4	5	6	7
1. Taboo Stroop Interference	–	0.07	−0.13	−0.14	−0.19	0.21	0.46 **
2. Neutral Valence		–	0.44 **	0.80 **	0.21	0.29	0.01
3. Taboo Valence			–	0.34 *	0.76 **	0.06	−0.17
4. Neutral Arousal				–	0.30	0.16	−0.16
5. Taboo Arousal					–	−0.12	−0.26
6. Neutral Familiarity						–	0.53 **
7. Taboo Familiarity							–

* $p < 0.05$; ** $p < 0.01$.

2. Experiment 2

2.1. Methods

Participants were 40 young (*M* = 20.5 years) and 40 older adults (*M* = 72.9 years) resembling those in Experiment 1. Snellen acuity was collected for 20 young and all 40 of the older adults. Snellen acuity was better for young (*M* = 25.0, *SD* = 5.13) than older adults (*M* = 40.9, *SD* = 9.26), $t(58) = 7.12$, but this age difference did not complicate the conclusions in MacKay *et al.* [1] because the size of the taboo Stroop interference effect (percent difference in mean color naming times for taboo *vs*. neutral words) did not correlate reliably with Snellen acuity across all participants in Experiment 2, ($r = -0.09$), or separately for young ($r = 0.11$), or older ($r = -0.19$) adults.

2.2. Rating Procedures

Following the color naming and color recognition tasks in MacKay *et al.* [1], the participants used the same scales as in Experiment 1 to rate the words in the color naming task (shown in Table S4).

Table S4. Base-words in Experiment 2 with length in letters and mean post-experimental ratings by word, word type, rating type, and age.

Type	Item	Word Length	Mean Valence Rating		Mean Arousal Rating		Mean Familiarity Rating	
			Young	Older	Young	Older	Young	Older
Neutral	Sheep	5	4.58	6.20	3.03a	1.88	7.00	6.95
	Crow	4	4.38	5.93	4.05	2.95	6.93	6.93
	Bee	3	4.35	5.93	4.38	3.43	7.00	7.00
	Turtle	6	4.88	6.05	2.70	1.98	7.00	7.00
	Skunk	5	3.78	5.20	4.45	4.13	6.95	6.93
	Mule	4	4.23	5.98	3.73	3.00	6.80	6.80
	Boar	4	4.20	5.68	4.18	3.63	6.70	6.68
	Bear	4	4.68	6.10	3.73	3.18	7.00	6.98
	Deer	4	4.73	6.35	2.73	1.68	7.00	6.95
	Hawk	4	4.70	5.93	3.80	2.68	6.93	6.90
	Shark	5	4.38	5.70	4.35	4.05	6.95	6.95
	Mouse	5	4.48	5.70	3.60	3.23	7.00	6.95
Mean		4.42	4.44	5.89	3.73	2.98	6.94	6.92
SD		0.79	1.16	1.57	1.37	1.82	0.33	0.48
Taboo	Chink	5	2.03	2.58	6.08	5.68	5.88	6.10
	Shit	4	2.73	2.83	5.30	5.50	6.98	6.78
	Fag	3	1.53	1.88	6.35	5.88	6.90	6.63
	Nigger	6	1.28	1.58	6.60	6.35	6.75	6.75
	Bitch	5	2.45	2.85	5.70	5.58	6.95	6.70
	Dick	4	3.08	2.85	5.25	5.35	7.00	6.55
	Cunt	4	1.63	2.18	6.25	6.25	6.65	6.35
	Dyke	4	1.88	2.68	5.90	5.78	6.45	6.35
	Fuck	4	2.5	2.05	5.35	6.08	7.00	6.70
	Piss	4	2.92	2.63	5.33	5.48	7.00	6.78
	Queer	5	2.58	2.93	5.50	5.38	6.78	6.68
	Whore	5	1.90	2.33	5.85	5.65	6.78	6.63
Mean		4.42	2.21	2.44	5.79	5.74	6.76	6.58
SD		0.79	1.13	1.47	1.82	1.31	0.83	1.16

2.3. Results and Discussion

Mean color naming latencies and recognition memory scores appear in MacKay *et al.* [1]. Here we report only results involving post-experimental ratings for valence, arousal, and familiarity. Mean ratings for the three scales are shown by word and word type for young and older adults in Table S4 (with "calmness" ratings re-scaled as before, so that high scores reflect high levels of emotional arousal). Separate age (young *vs*. older) by word type (neutral *vs*. taboo) ANOVAs on the valence and arousal ratings yielded results consistent with those for naïve participants in MacKay and Ahmetzanov [3]: higher positive valence ratings for neutral (M = 5.11) than taboo words (M = 2.26), $F(1, 78) = 324.45$, $MSE = 0.88$, $p < 0.001$, $\eta_p^2 = .83$, and lower arousal ratings for neutral (M = 3.35) than taboo (M = 5.77) words, $F(1, 78) = 232.81$, $MSE = 0.76$, $p < 0.001$, $\eta_p^2 = 0.80$. In addition, older adults rated the words more positively than young adults (M's = 4.04 and 3.32, respectively), $F(1, 78) = 18.53$, $MSE = 1.11$, $p < .001$, $\eta_p^2 = 0.19$, with a significant age by word type interaction for valence, F (1, 78) = 16,89, $MSE = 0.88$, $p < 0.001$, $\eta_p^2 = 0.18$, such that the age difference in ratings occurred for neutral but not taboo words. Older adults rated the words as less arousing than young adults, F(1, 78) = 6.34, $MSE = .98$, $p = 0.014$, $\eta_p^2 = 0.08$, and again age and word type interacted, $F(1, 78) = 6.44$, $p = 0.013$, $MSE = 0.76$, $\eta_p^2 = 0.08$, such that older adults rated the neutral set of words as less arousing than young adults, while the age difference for taboo words was non-significant (see Table S4). As in Experiment 1, familiarity ratings were higher for neutral (M = 6.92) than taboo words (M = 6.67), $F(1, 78) = 16.17$, $MSE = 0.16$, $p < 0.001$, $\eta_p^2 = 0.17$. However unlike Experiment 1, older adults did not provide higher familiarity ratings than young adults.

Because of these age differences in post-experimental valence ratings, we again wondered whether the age-invariance in taboo Stroop interference observed in MacKay *et al.* [1] reflected participant-specific perceptions of valence, arousal, or familiarity (measured post-experimentally). As before, we explored this issue by examining bivariate correlations between mean rating scores on each dimension for each word type and the degree of taboo Stroop interference within each age group. These correlations appear in Tables S5 (young adults) and S6 (older adults). Unlike Experiment 1, there were no significant correlations between taboo Stroop interference and any of the ratings for young or older adults. As before, concerns about ceiling effects in the familiarity ratings led us to re-calculate this correlation excluding the older participants who gave all of the taboo words the highest possible rating (n = 24). This time, the size of the correlation between mean taboo familiarity and taboo Stroop interference was larger, $r(16) = 0.42$, $p = 0.10$, but remained non-significant.

Table S5. Bivariate correlations of taboo Stroop interference, valence, arousal, and familiarity ratings for young adults in Experiment 2.

Variable	1	2	3	4	5	6	7
1. Taboo Stroop Interference	−	−0.05	0.13	0.23	0.03	0.00	−0.04
2. Neutral Valence		−	0.20	−0.71 **	−0.26	0.06	−0.17
3. Taboo Valence			−	−0.21	−0.63 **	0.05	−0.15
4. Neutral Arousal				−	0.38 *	−0.17	0.11
5. Taboo Arousal					−	−0.13	−0.06
6. Neutral Familiarity						−	0.61 **
7. Taboo Familiarity							−

* $p < 0.05$; ** $p < 0.01$.

Table S6. Bivariate correlations of taboo Stroop interference, valence, arousal, and familiarity ratings for older adults in Experiment 2.

Variable	1	2	3	4	5	6	7
1. Taboo Stroop Interference	−	0.19	0.05	−0.02	0.08	−0.08	0.17
2. Neutral Valence		−	0.09	−0.71 **	0.18	−0.17	−0.24
3. Taboo Valence			−	−0.08	−0.55 **	−0.08	−0.07
4. Neutral Arousal				−	0.00	0.10	0.29
5. Taboo Arousal					−	0.07	0.09
6. Neutral Familiarity						−	0.64 **
7. Taboo Familiarity							−

* $p < 0.05$; ** $p < 0.01$.

3. General Discussion

Post-experiment ratings in Experiments 1 and 2 exhibited age differences and age by word type interactions, but only the main effects of word type on valence, arousal, and familiarity; the main effects of age on valence and arousal; and the age by word type interaction on valence replicated across both experiments. Because MacKay *et al.* [1] found age invariance in taboo Stroop interference, it is possible that the age differences in the perceived emotionality of the material may have suppressed an age difference in interference. If this were the case, we predicted correlations between the post-experiment ratings and the magnitude of the taboo Stroop effect. However we failed to find consistent correlations in either age group. This renders it unlikely that age differences in perceived valence, arousal, or familiarity underlie the age constancy in taboo Stroop interference observed in Experiments 1 and 2 (see [1]). The post-experimental ratings for valence, arousal, and familiarity do not therefore complicate interpretation of the age-linked findings in MacKay *et al.* [1]. The age differences in Snellen acuity in Experiments 1 and 2 likewise did not complicate the conclusions in MacKay *et al.* [1] because in neither experiment did the size of the taboo Stroop interference effect correlate reliably with acuity scores.

References

1. MacKay, D.G.; Johnson, L.W.; Graham, E.R.; Burke, D. Aging, emotion, attention and binding in the taboo Stroop task: Data and theories, submitted.
2. Siegrist, M. Effects of taboo words on color-naming performance on a Stroop test. *Percept. Motor Skills* 1995, *81*, 1119–1122.
3. MacKay, D.G.; Ahmetzanov, M.V. Emotion, memory, and attention in the taboo Stroop paradigm: An experimental analog of flashbulb memories. *Psycholog. Sci.* **2005**, *16*, 25–32.

© 2015 by the authors; licensee MDPI, Basel, Switzerland. This article is an open access article distributed under the terms and conditions of the Creative Commons Attribution license (http://creativecommons.org/licenses/by/4.0/).
